# Impacts of Hyperglycemia on Epigenetic Modifications in Human Gingival Fibroblasts and Gingiva in Diabetic Rats

**DOI:** 10.3390/ijms252010979

**Published:** 2024-10-12

**Authors:** Kento Kojima, Nobuhisa Nakamura, Airi Hayashi, Shun Kondo, Megumi Miyabe, Takeshi Kikuchi, Noritaka Sawada, Tomokazu Saiki, Tomomi Minato, Reina Ozaki, Sachiko Sasajima, Akio Mitani, Keiko Naruse

**Affiliations:** 1 Department of Periodontology, School of Dentistry, Aichi Gakuin University, Suemori-dori, Chikusa-ku, Nagoya 4648651, Japan; ag213d09@dpc.agu.ac.jp (K.K.); ag233d16@dpc.agu.ac.jp (A.H.); shunk@dpc.agu.ac.jp (S.K.); tkikuchi@dpc.agu.ac.jp (T.K.); nsawada@dpc.agu.ac.jp (N.S.); minita@dpc.agu.ac.jp (A.M.); 2 Department of Internal Medicine, School of Dentistry, Aichi Gakuin University, Suemori-dori, Chikusa-ku, Nagoya 4648651, Japan; mmiyabe@dpc.agu.ac.jp (M.M.); saiki@dpc.agu.ac.jp (T.S.); minato19@dpc.agu.ac.jp (T.M.); reinaozk@dpc.agu.ac.jp (R.O.); sachiko3@dpc.agu.ac.jp (S.S.); narusek@dpc.agu.ac.jp (K.N.)

**Keywords:** periodontal tissue, matrix metalloproteinases, epigenome, histone modifications

## Abstract

Periodontal disease is considered one of the diabetic complications with high morbidity and severity. Recent studies demonstrated the involvement of the epigenome on diabetic complications. Histone modifications change chromatin architecture and gene activation. Histone modifications have been reported to alter chromatin structure and regulate gene transcription. In this study, we investigated the impacts of H3 lysine 4 trimethylation (H3K4me3) and specific histone methyltransferases of H3K4 methylation, su(var)3-9, enhancer-of-zeste, and trithorax domain 1A (SETD1A) on periodontal tissue affected by the diabetic condition. We observed the increase in H3K4me3 and SETD1A in gingival tissue of diabetic rats compared with the normal rats. Cultured human fibroblasts (hGFs) confirmed a high glucose-induced increase in H3K4me3 and SETD1A. We further demonstrated that high glucose increased the gene expression of *matrix metalloproteinase* (*MMP*) *1* and *MMP13*, which were canceled by sinefungin, an SETD1A inhibitor. Our investigation suggests that diabetes triggers histone modifications in the gingival tissue, resulting in gingival inflammation. Histone modifications may play crucial roles in the development of periodontal disease in diabetes.

## 1. Introduction

Periodontal disease is a chronic inflammatory disease in which periodontal tissue is destroyed by bacterial infection. Periodontal tissue constituent cells have been consistently exposed to various risk factors, including bacterial factors such as plaque and systemic factors such as lifestyle-related diseases [1,2]. Systemic diseases such as diabetes, leukemia, and osteoporosis are thought to affect periodontal tissue [3,4]. In particular, the relationship between diabetes and periodontal disease has been well investigated. Diabetes is a chronic, metabolic disease characterized by elevated levels of blood glucose. The glucose level criteria for diagnosis of diabetes are fasting plasma glucose level ≥ 126 mg/dL, 2 h plasma glucose level ≥ 200 mg/dL on oral glucose tolerance test, or plasma glucose level ≥ 200 mg/dL at any time [5]. Type 1 diabetes is a chronic condition in which the pancreas produces little or no insulin through autoimmune. Type 2 diabetes is the most common type, usually in adults, which occurs when the body becomes resistant to insulin or does not make enough insulin. It is known that periodontal disease has high morbidity and susceptibility to severe disease in both type 1 and type 2 diabetes [6,7,8,9]. In the grading of periodontitis formulated by the 2017 World Workshop on the Classification of Periodontal and Peri-Implant Diseases and Conditions, diabetes is one of the risk factors that modifies the grade of periodontitis [10].

Hyperglycemia causes epigenetic changes in fully differentiated cells, resulting in cellular dysfunction that contributes to pathology [11]. Epigenetic modifications such as DNA methylation, histone modifications, and microRNAs (miRNAs) can affect transcriptional activity without altering the DNA sequence [12]. They are susceptible to environmental factors such as nutrition, chemicals, stress, drugs, and infections, and are essential for the control of all biological processes [13]. Epigenetic changes have also been reported to be associated with inflammatory diseases, including obesity and type 2 diabetes, and chronic hyperglycemia affects epigenetic modifications that can lead to various chronic complications [14].

Histone methylation is a critical post-translational modification that regulates gene expression by modifying the chromatin structure. It involves the addition of methyl groups (–CH_3_) to specific lysine or arginine residues on histone proteins, typically on histone H3 or H4. This modification can either activate or repress transcription, depending on the site and context of the methylation. Methylation is mediated by histone methyltransferases (HMTs), which transfer methyl groups from S-adenosyl methionine (SAM) to histones. Histone demethylases (HDMs) can remove these groups. Histone methylation is involved in various biological processes such as the development, differentiation, and maintenance of cellular identity. Histone proteins undergo post-translational modification in different ways, which impacts their interactions with DNA [15,16]. In particular, the trimethylation of histone H3 lysine 4 (H3K4me3) is associated with transcriptional start sites and has been proposed to regulate transcription initiation [17,18].

Previous studies have shown that H3K4me3 is produced in diabetes models. H3K4me3 is a chromatin modification at the transcriptional initiation site of active eukaryotic genes from yeast to humans and reflects the amount of transcription [19,20]. HMT is a histone-modifying enzyme that transfers a methyl group to a lysine residue of histone H3. HMTs are divided into two categories: SET domain-containing enzymes and non-SET domain enzymes [21,22,23]. These have become important because of their diverse roles in diseases such as diabetes complications, cancer, and atherosclerosis [24,25]. Su(var)3-9, enhancer-of-zeste, and trithorax domain 1A (SETD1A) is known to be a specific histone methyltransferase of H3K4 metylation. Matrix metalloproteinase (MMP) and tissue inhibitors of metalloproteinases (TIMPs) are regulated to prevent excessive MMP activity in diabetes [26,27]. Hyperglycemia regulates the gene expression of inflammatory cytokines and matrix metalloproteinases (MMPs) by increased H3K4me3 expression [28,29]; however, the underlying mechanism has not yet been elucidated. Since MMPs, especially fibrous collagenases such as MMP1 and MMP13, have been associated with periodontal disease, we investigated the effects of hyperglycemia on histone-methylated proteins and histone methyltransferases, and whether histone methylation affects tissue destruction-related genes on periodontal tissue due to diabetes.

The aim of the present study was to investigate whether histone modifications in the gingival tissue were induced in diabetic rats. Furthermore, the expression of genes associated with tissue destruction, such as MMPs, in hyperglycemic human gingival fibroblasts was investigated via histone modifications.

## 2. Results

### 2.1. Body Weight, Blood, and Alveolar Bone Evaluation

**Figure 1:**
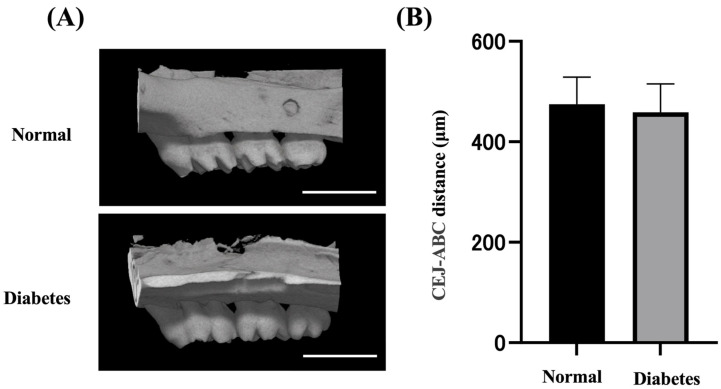
Comparison of alveolar bone between control group and diabetic group. (**A**) Three-dimensional reconstructed image of alveolar bone using microcomputed tomography (µCT). Scale = 3000 µm. (**B**) Alveolar bone was assessed by µCT of the distance from the cement–enamel junction (CEJ) to the alveolar bone crest (ABC). The results are presented as the mean ± S.E.M. (*n* = 6).

Diabetic rats had significantly lower body weight and higher blood glucose levels than normal rats (*n* = 6) (Table 1). Alveolar bone samples from the left maxillary molars of the rats were collected, and microcomputed tomography was performed to confirm the state of alveolar bone 2 weeks after the induction of diabetes (Figure 1A). Compared with the normal group, no significant bone changes were observed in the diabetic group (Figure 1B).

### 2.2. Diabetes Upregulates Histone Methylation and SETD1A in the Gingival Tissues

**Figure 2:**
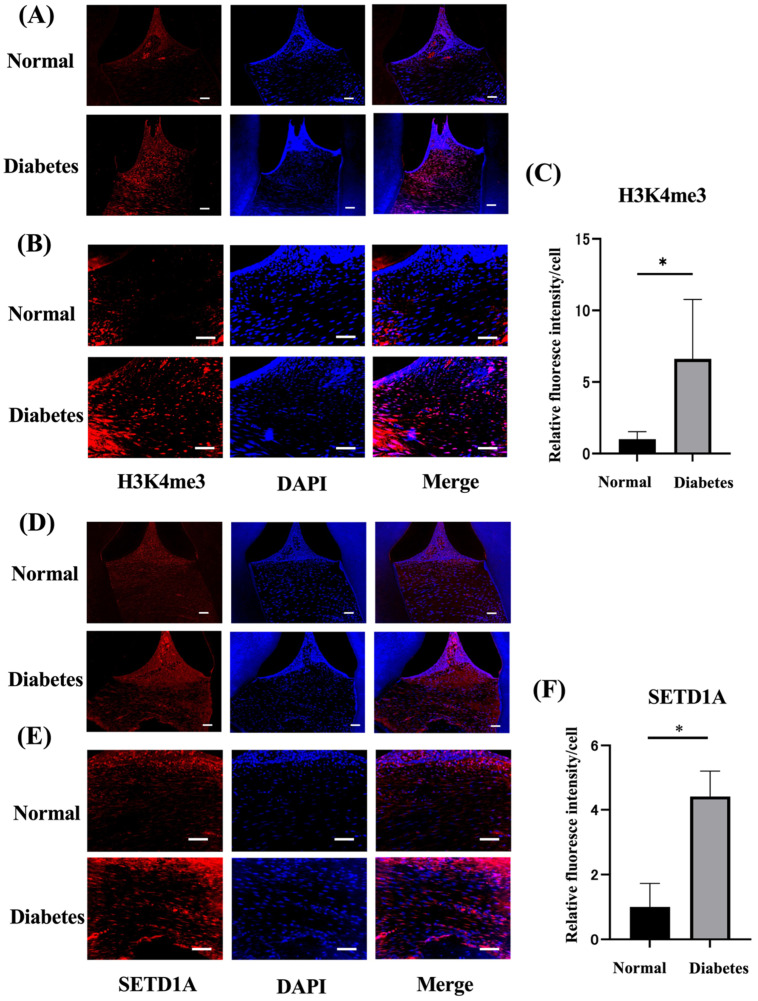
Expression of H3K4me3 and SETD1A in periodontal tissue in the control and diabetes groups. The expression of local H3K4me3 and SETD1A in gingival tissues was visualized by immunohistochemical staining. The immunofluorescence staining of nuclei stained with 4′,6-diamidino-2-phenylindole (DAPI, blue) and H3K4me3 (red), SETD1A (red) was performed. (**A**) Low-power images of H3K4me3 in the periapical maxillary second molar gingiva of control and diabetic rats. Scale bar = 50 µm (×20). (**B**) High-magnification images of H3K4me3. Scale bar = 50 µm (×40). (**C**) Quantitative measurement of H3K4me3 expression per cell was performed using Image J (*n* = 4). (**D**) Low-power image of SETD1A in the periapical maxillary second molar gingiva of normal and diabetes rats. Scale bar = 50 µm (×20). (**E**) High-magnification images of SETD1A. Scale bar = 50 µm (×40). (**F**) Quantitative measurement of SETD1A expression per cell was performed using Image J. * *p* < 0.05.

To investigate the expression of histone methylation and histone methyltransferases in the gingival tissues of rats with diabetes, gingival tissues were collected 4 weeks after the induction of diabetes. The protein expression of H3K4me3 and the histone modification enzyme SETD1A in periodontal tissues were assessed by the immunofluorescence staining of tissue sections. As shown in Figure 2A,B, the expression of H3K4me3 was significantly higher (6.3 times) than that in control rats (Figure 2C). The expression of SETD1A was also significantly increased (4.2-fold) compared to control rats (Figure 2D,E). Quantitative evaluation using ImageJ2 (ver.2.15.0: National Institutes of Health (NIH), Bethesda, MD, USA) revealed that the expression of H3K4me3 and SETD1A in periodontitis rats was significantly increased by 2.0 times compared with control rats. 

### 2.3. Expression of Histone-Methylated Proteins and SETD1A and the Effects of Histone Methyltransferase (HMT) Inhibitor in High-Glucose-Stimulated hGFs

**Figure 3:**
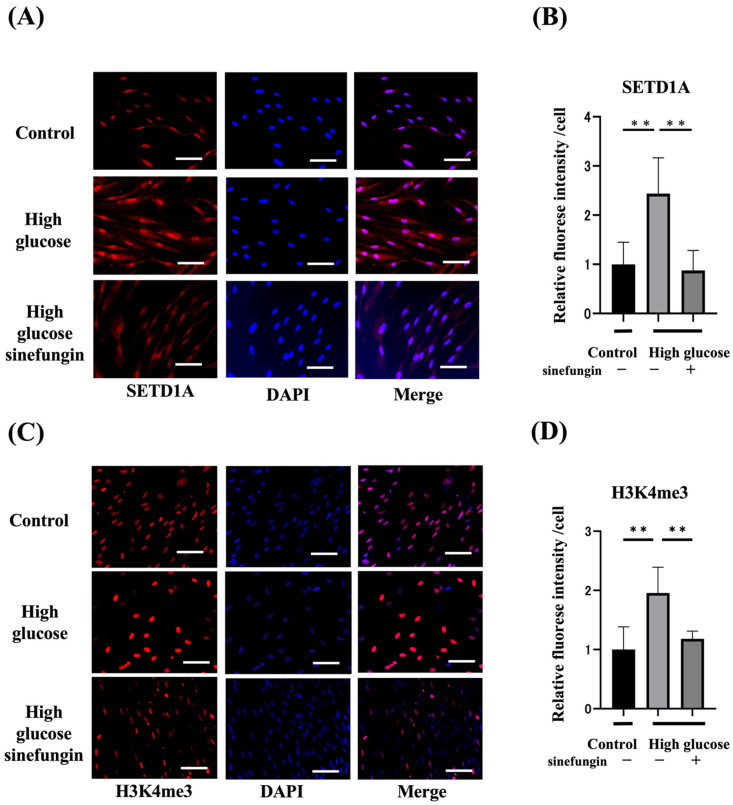
Immunohistochemical staining revealed changes in the expression of histone-methylated proteins (H3K4me3) and histone methyltransferases (SETD1A) owing to high glucose levels. The immunofluorescence staining of the nuclei with DAPI (blue), H3K4me3 (red), and SETD1A (red) was performed. (**A**) H3K4me3 stained images of control, high glucose, and high glucose with sinefungin. Scale bar = 50 µm (×40). (**B**) ImageJ software (*n* = 6) was used to quantitatively measure the H3K4me3 expression per cell. (**C**) SETD1A stained images of control, high glucose, and high glucose with sinefungin. Scale bar = 50 µm (×40). (**D**) ImageJ software (*n* = 6) was used to quantitatively measure the SETD1A expression per cell. ** *p* < 0.01.

We inhibited SETD1A in hGFs using sinefungin (2 µm) [30], a non-selective HMT inhibitor, to clarify the effect of SETD1A on the expression of histone-methylated proteins induced by high glucose (27.5 mM) and normal glucose (5.5 mM) in hGFs. Immunohistochemical staining revealed that high glucose increased the expression of SETD1A in hGFs, which was suppressed by sinefungin (Figure 3A,B). High glucose levels increased the fluorescence intensity of H3K4me3 by approximately 2.2 times, while sinefungin suppressed the expression of H3K4me3 (Figure 3C,D).

### 2.4. Sinefungin Regulates Gene Expression of MMPs and Improves the MMP-TIMP Ratio (MMP/TIMP) in hGFs

**Figure 4:**
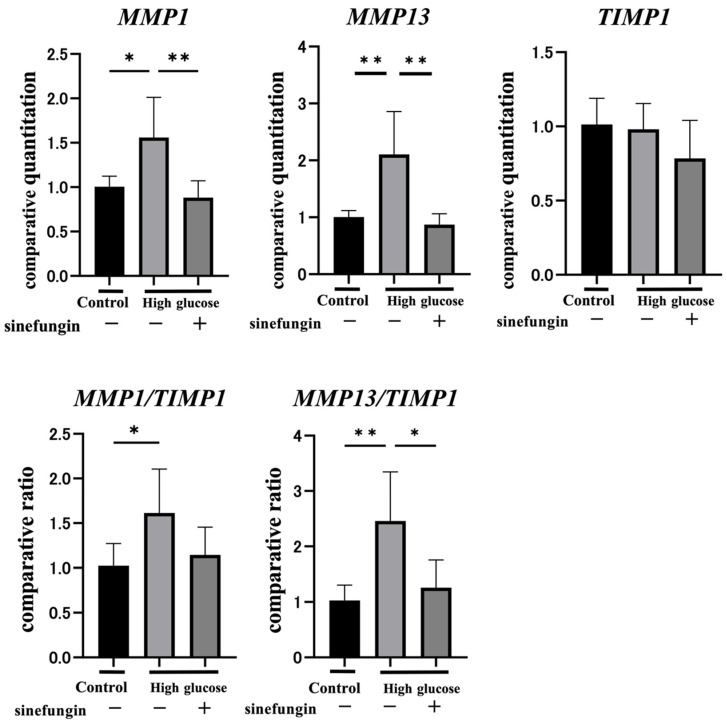
The expression of MMPs and TIMPs in cells cultured with D-glucose (27.5 mM) was inhibited by sinefungin. The hGFs were cultured with D-glucose (27.5 mM) and inhibited by sinefungin. The mRNA expression levels of MMP1, MMP13, and TIMP1 were analyzed using real-time polymerase chain reaction. Results are presented as the mean ± S.E.M. (*n* = 6). * *p* < 0.05. The ratio was calculated and analyzed using comparative quantitative values of MMP and TIMP (*n* = 6). * *p* < 0.05, ** *p* < 0.01.

High glucose significantly increased the expressions of *MMP1* and *MMP13* in hGFs by 1.6 times and 2.1 times, respectively (Figure 4). In contrast, the gene expression of TIMP1 was not changed between the normal and high glucose groups. To elucidate the role of SETD1A in the expression of *MMP1*, *MMP13*, and *TIMP1* in response to high glucose levels, sinefungin was used to inhibit *SETD1A* expression in hGFs. The inhibition of *SETD1A* by sinefungin eliminated the high glucose-increased *MMP1* and *MMP13* gene expression. Sinefungin did not affect *TIMP1* expression in high glucose conditions. When the *MMP/TIMP* ratio was confirmed, high glucose significantly increased *MMP1/TIMP1* by 1.6 times and *MMP13/TIMP1* by 2.2 times, which were normalized by sinefungin (Figure 4).

## 3. Discussion

In this study, we investigated the roles of histone proteins and methyltransferases in hyperglycemia. Diabetic rats showed increased H3K4me3 and SETD1A expression in gingival tissues. In hGFs, high glucose levels led to the increased expression of H3K4me3 and SETD1A. Furthermore, the HMT inhibitor, sinefungin, regulated the expression of *MMPs* and *MMP/TIMP* by downregulating the production of H3K4me3 and SETD1A in hGFs. These results suggest the involvement of histone methylation in periodontal disease in diabetes.

We demonstrated that the diabetes group showed histone modifications compared with the control group four weeks after the induction of diabetes. Our results reveal significant increases in H3K4me3 and SETD1A levels in the gingival tissues of the diabetic group compared with those in the control group. These results reveal that hyperglycemia-induced changes in gingival tissue are associated with an increase in H3K4me3 mediated by SETD1A. Previous studies have reported that HMTs, such as SET1 and MLL, contribute to diabetes-related vascular complications and diabetic wound healing failure by causing an increase in H3K4me3 in specific gene promoters; our results are consistent with these reports [31,32].

MMPs are a family of zinc-dependent endopeptidases that are the most important enzymes involved in extracellular matrix (ECM) degradation. They play key roles in the restructuring of physiological and pathological tissues [33]. TIMP is an endogenous matrix metalloproteinase (MMP) inhibitor [34]. Fibrous collagenases such as MMP1 and MMP13 have been associated with diabetic complications and periodontal disease. Previous studies have reported that glucose neurotoxicity is caused by the increased expression of MMP13 with the formation of H_2_O_2_ due to hyperglycemia [35]. The function of the MMP/TIMP balance in diabetic wound healing insufficiency is affected by hyperglycemia [36]. The increase in the MMP/TIMP ratio indicates a persistent imbalance in the degradation and synthesis of ECM in gingival tissues affected by periodontitis and is responsible for increased tissue destruction in periodontitis [37]. In addition, it has been reported that the MMP/TIMP ratio increases due to an increase in MMP and the inactivity of TIMP in periodontal ligaments, to which corrective force is applied in diabetic model animals, and that increased MMP/TIMPs are an important process that increases tissue destruction in diabetic periodontitis [38]. In this study, a significant change in MMP due to high glucose levels was observed. However, the effect on TIMP1 expression was minor. In addition, MMP/TIMP expression increased, as did the expression of MMP genes, and this change was related to H3K4me3 mediated by SETD1A.

The mechanisms by which high glucose induce histone methylation have not yet been elucidated. The TGF-β signaling pathway, activated by high glucose, stimulates the histone machinery: after being induced by TGF-β1, the methyltransferase SET7/9 is enriched at fibrosis gene promoters. As a result of this enrichment, positive chromatin markers such as H3K4me3 increase and repressive markers such as H3K9me3 decrease at fibrosis gene promoters [39,40,41]. In diabetes, fumaric acid and succinic acid are synthesized as metabolites of the TCA cycle. These metabolic changes inhibit histone lysine demethylases (KDMs), which have been found to promote histone methylation [42]. It has also been reported that α-ketoglutarate regulates KDMs involved in histone modifications that suppress the gene expressions such as H3K9me3 and H3K27me3 [43], suggesting that metabolites in the TCA cycle may affect histone modifications [44,45]. Further study is required to elucidate the molecular mechanisms. 

The limitation of our study is that we did not investigate the involvement of insulin resistance in histone methylation because we used STZ-induced type 1 diabetic models. Primary cell cultures taken directly from the two groups of rats could not be used due to infection or purity. Further studies are required to address this issue.

## 4. Materials and Methods

### 4.1. Animals

Sprague Dawley rats (*n* = 12) were purchased from Japan SLC (Shizuoka, Japan), freely fed standard food and water, and housed in individual cages (260 mm × 382 mm × 200 mm, 3 per cage) under 12 h of a light and dark cycle at a controlled temperature (24.0 ± 1.0 °C). This study was approved by the Aichi Gakuin University Institutional Animal Control and Use Committee (AGUD464), and all animal experiments were conducted in accordance with national guidelines for the protection of animals and relevant laws.

### 4.2. Induction of Diabetes

Male Sprague Dawley rats aged 6 weeks were divided into two groups: control and diabetic rats. Diabetes was induced by intraperitoneally injecting STZ (60 mg/kg; Fujifilm Wako Corporation, Tokyo, Japan), and control rats were injected with saline. 

### 4.3. Tissue Collection

Four weeks after the induction of diabetes, the rats were sacrificed using CO_2_, and samples were prepared. For immunohistological staining and micro-CT analyses, maxillary bones with gingival tissues attached on both sides were fixed with 4% paraformaldehyde (Fujifilm Wako Corporation, Tokyo, Japan).

### 4.4. Microcomputed Tomography

A detailed protocol was carried out according to our previously published study [46,47]. Briefly, rat maxillary bones fixed with 4% paraformaldehyde were subjected to microcomputed tomography (RmCT; Rigaku Corporation, Tokyo, Japan). Three-dimensional reconstruction was performed using the TRI/3D-BON (Ratoc Corporation, Tokyo, Japan). The distance from the mesial buccal cemento-enamel junction (CEJ) to the alveolar bone crest (ABC) of the second molar was measured as a reference for bone height. Two skilled researchers blindly calculated bone resorption as the distance from the buccal cemento-enamel junction to the apex of the alveolar bone.

### 4.5. Immunohistological Evaluation of Periodontal Tissue

For immunohistochemical staining, the maxillary bone with the attached bilateral gingival tissue was fixed in 4% paraformaldehyde. Periodontal tissue, including fixed alveolar bone, was demineralized with 10% ethylenediaminetetraacetic acid for 5 weeks, embedded in paraffin, and cut into 5 µm sections. Sections were stained with anti-H3K4me3 (Cell Signaling Technology, Danvers, MA, USA), Su(var)3-9, enhancer-of-zeste, and trithorax domain 1A (SETD1A) (Abcam Corporation, Cambridge, UK). Subsequently, the periodontal tissue was stained with 4′,6-diamidino-2-phenylindole (DAPI), and Alexa Fluor 594-conjugated goat anti-rabbit IgG (Thermo Fisher Scientific, Waltham, MA, USA) as the secondary antibodies for 1 h at 4 °C. Images were captured using a fluorescence microscope (BZ-X700, Keyence, Osaka, Japan). The fluorescence intensity per cell count in the tissue images was calculated using the ImageJ software.

### 4.6. Cell Culture

Human gingival fibroblasts (hGFs) were obtained from ScienCell Research Laboratories (Carlsbad, CA, USA). The hGFs were cultured in Dulbecco’s modified Eagle’s medium (Fujifilm Wako) supplemented with 10% fetal bovine serum (GIBCO Laboratories Inc., Grand Island, NY, USA). The cells were cultured at 37 °C in an incubator containing 5% CO_2_. Cells were incubated with glucose (27.5 mM) and 2 µM sinefungin (Abcam Corporation, Cambridge, UK) for 7 days.

### 4.7. Immunohistochemical Staining of Gingival Fibroblasts

The cells were fixed with 4% paraformaldehyde. For the immunohistochemical staining of cultured fibroblasts, hGF cells were fixed and incubated with primary anti-rabbit H3K4me3 (Cell Signaling Technology, Danvers, MA, USA) and anti-rabbit SETD1A (Abcam Corporation, Cambridge, UK) antibodies in PBS (Fujifilm Wako, Tokyo, Japan). Subsequently, the hGFs were incubated with 4′,6-diamidino-2-phenylindole (DAPI), and Alexa Fluor 594-conjugated goat anti-rabbit IgG as the secondary antibodies for 1 h at 4 °C. The fluorescence intensity per cell was calculated using the ImageJ software.

### 4.8. Gene Expression Analyses

A detailed protocol was carried out according to our previously published study [48]. Briefly, total RNA was extracted from the hGFs, and the total RNA purity and concentration were determined. Reverse transcription was performed, and quantitative polymerase chain reaction (qPCR) was performed using TaqMan Real-Time PCR Master Mix and TaqMan Assay probes (Thermo Fisher Scientific, Waltham, MA, USA) with SETD1A (Hs00322315_m1), MMP1 (Hs00899658_m1), MMP13 (Hs00233992_m1), TIMP1 (Hs01092512_g1) as target gene, and beta-actin (Human ACTB4352935E) as inner control gene, were used (Thermo Fisher Scientific, Waltham, MA, USA). Relative mRNA levels were calculated using the ΔΔCt method. Real-time quantitative PCR was performed using a Light Cycler 480 system (Roche Diagnostics, Basel, Switzerland). 

### 4.9. Statistical Analysis

Data are expressed as mean ± S.E.M. Differences between the two groups were evaluated using an unpaired two-tailed Student’s *t*-test, unless otherwise noted. Datasets containing more than two groups were evaluated using analysis of variance (one-way ANOVA), followed by Bonferroni correction for multiple comparisons. A *p*-value less than 0.05 was set as statistically significant.

## 5. Conclusions

**Figure 5:**
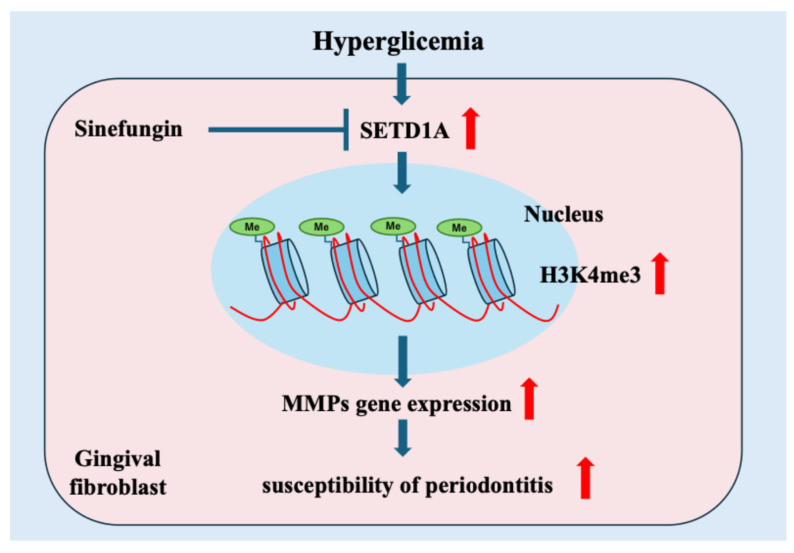
Schematic of mechanisms of increasing susceptibility to periodontal disease in diabetes. Hyperglycemia-induced upregulation of SETD1A increases H3K4me3 levels, which in turn leads to elevated expression of MMPs.

We discovered that diabetes causes histone modifications of H3K4me3 via the upregulation of SETD1A in gingival tissues. These histone modifications may be involved in periodontal tissues in diabetes, increasing susceptibility to periodontal disease in individuals with diabetes (Figure 5).

## Figures and Tables

**Table 1:** Characteristics of the normal and diabetic rats.

Variable	Normal Rats	Diabetes Rats
Body weight (g)	385.8 ± 22.1	276.7 ± 16.2 **
Blood glucose (mmol/L)	6.1 ± 0.9	26.4 ± 7.3 **

Values are given as mean ± S.E.M. (*n* = 6). ** *p* < 0.01 vs. normal rats.

## Data Availability

The data presented in this study are available on request from the corresponding author.
